# The complete mitochondrial genome of *Sinojackia microcarpa*: evolutionary insights and gene transfer

**DOI:** 10.1186/s12864-025-11633-7

**Published:** 2025-05-06

**Authors:** Tailin Zhong, Shijie Huang, Rongxiu Liu, Juan Zhuo, Haifei Lu, Chunlin Gan, Jun Fu, Qixia Qian

**Affiliations:** 1 College of Urban Construction of Zhejiang Shuren University, Key Laboratory of Pollution Exposure and Health Intervention of Zhejiang Province, Zhejiang Shuren University, Shaoxing, Zhejiang People’s Republic of China; 2 National Key Laboratory for Development and Utilization of Forest Food Resources, Zhejiang A&F University, Hangzhou, 311300 China; 3 Key Laboratory of Bamboo Science and Technology of Ministry of Education, Bamboo Industry Institute, Zhejiang A&F University, Hangzhou, 311300 China; 4 Lishan Forest Farm, Xin’gan County, Xin’gan, Jiangxi People’s Republic of China; 5 State-owned Paiyangshan Forest Farm in Guangxi Zhuang Autonomous Region, Ningming, Guangxi People’s Republic of China; 6 College of Landscape Architecture, Zheiiang A&F University, Hangzhou, 311300 China

**Keywords:** *Sinojackia microcarpa*, Mitochondria genome, Gene transfer, Phylogenetic analysis

## Abstract

**Background:**

As a dicotyledonous plant within the Styracaceae family, *Sinojackia microcarpa* (*S. microcarpa*) is notable for its library-shaped fruit and sparse distribution, serving as a model system for studying the entire tree family. However, the scarcity of genomic data, particularly concerning the mitochondrial and nuclear sequences of *S. microcarpa*, has substantially impeded our understanding of its evolutionary traits and fundamental biological mechanisms.

**Results:**

This study presents the first complete mitochondrial genome sequence of *S. microcarpa* and conducts a comparative analysis of its protein-encoding genes across eight plant species. Our analysis revealed that the mitochondrial genome of *S. microcarpa* spans 687,378 base pairs and contains a total of 59 genes, which include 37 protein-coding genes (PCGs), 20 transfer RNA (tRNA) genes, and 2 ribosomal RNA (rRNA) genes. Sixteen plastid-derived fragments strongly linked with mitochondrial genes, including one intact plastid-related gene (*rps7*), were identified. Additionally, Ka/Ks ratio analysis revealed that most mitochondrial genes are under purifying selection, with a few genes, such as *nad9* and *ccmB*, showing signs of relaxed or adaptive evolution. An analysis of twenty-nine protein-coding genes from twenty-four plant species reveals that *S. microcarpa* exhibits a closer evolutionary relationship with species belonging to the genus Camellia. The findings of this study provide new genomic data that enhance our understanding of *S. microcarpa*, and reveal its mitochondrial genome’s evolutionary proximity to other dicotyledonous species.

**Conclusions:**

Overall, this research enhances our understanding of the evolutionary and comparative genomics of *S. microcarpa* and other plants in the Styracaceae family and lays the foundation for future genetic studies and evolutionary analyses in the Styracaceae family.

**Supplementary Information:**

The online version contains supplementary material available at 10.1186/s12864-025-11633-7.

## Background

The mitochondrial genome is a crucial genetic material in eukaryotic cells, garnering significant attention due to its central role in energy metabolism [1–3]. Compared to the nuclear genome, the mitochondrial genome exhibits distinctive characteristics such as a smaller genome size, maternal inheritance, a circular structure, and a relatively rapid evolutionary rate [3–5]. It plays a pivotal role in cellular energy production and in regulating apoptosis, calcium ion homeostasis, and other vital cellular functions [6–9]. In recent years, with the advancement of high-throughput sequencing technologies, research on the mitochondrial genome has made significant progress in fields such as evolutionary biology, population genetics, and disease diagnosis [10–12]. Additionally, the diversity and structural variation of the mitochondrial genome offers new insights into understanding biological adaptive evolution [13–15]. Therefore, studying the mitochondrial genome sheds light on its functional mechanisms and broad implications for research on biological evolution and adaptation [16–18].

*Sinojackia microcarpa* Tao Chen & G. Y. Li, a deciduous large shrub in the Styracaceae family, belongs to the Sinojackia genus. It is a rare and endangered species endemic to Zhejiang Province, China, with known distributions in the Lin’an and Jiande areas [19]. Endangered plants typically refer to plant groups that can no longer reproduce or sustain their populations under natural conditions due to various endangering factors. The population size of these plants has decreased to a critical level, posing a risk of extinction [20]. China harbors a multitude of endemic species, among which a significant number of wild plants are threatened. It is estimated that over 4,000 species are at risk, accounting for 15–20% of the total species [21, 22]. In previous work, we assembled the chloroplast genome of *S. microcarpa* and identified *rbcL* as a suitable DNA barcode marker for analyzing the phylogenetic relationships within the Styracaceae family [23]. Additionally, the genetic diversity of *S. microcarpa* was analyzed using simple sequence repeat (SSR) markers [24, 25]. However, our work is still insufficient. To further study and understand *Sinojackia microcarpa*, we have assembled and annotated its mitochondrial genome, aiming to gain a better understanding of this species and improve the conservation of these valuable biological resources. These biological resources are valuable for scientific research and may contribute to a better understanding of biological mechanisms, with potential applications in various fields.

The formation, development, endangerment, and extinction of species encompass the entire interaction between a species and its environment, driven by both intrinsic genetic factors and external ecological conditions [26–28]. With advancements in research techniques and methodologies, our understanding of species formation and its underlying mechanisms has significantly deepened, surpassing that of earlier studies. Numerous plant mitochondrial genomes, including those of economically important crops such as rice (*Oryza sativa*), wheat (*Triticum aestivum*), and tobacco (*Nicotiana tabacum*), have been sequenced and annotated [23, 29, 30]. Mitochondrial DNA studies in plants, such as in *Pinus edulis*, help assess genetic diversity and population structure, providing valuable insights for developing targeted conservation strategies [31]. The study of mitochondrial genomes is crucial for understanding the evolutionary relationships, genetic diversity, and adaptive mechanisms of species. Research on the mitochondrial genome of *S. microcarpa* will enhance our understanding of its distinct biological mechanisms [32, 33]. No nuclear or mitochondrial genome sequences for *S. microcarpa* have been made available, leaving critical evolutionary traits and biological mechanisms, such as its unique fruit morphology and factors contributing to its endangered status, largely unexplored. In this study, we present the first complete mitochondrial genome of *S. microcarpa*, comparing its features with those of other sequenced angiosperms. Our findings provide new insights into the evolutionary characteristics of the *S. microcarpa* mitochondrial genome, enhancing our understanding of its biological complexity.

## Results

### Assembly and features of the S. microcarpa mitochondrial genome

**Fig. 1:**
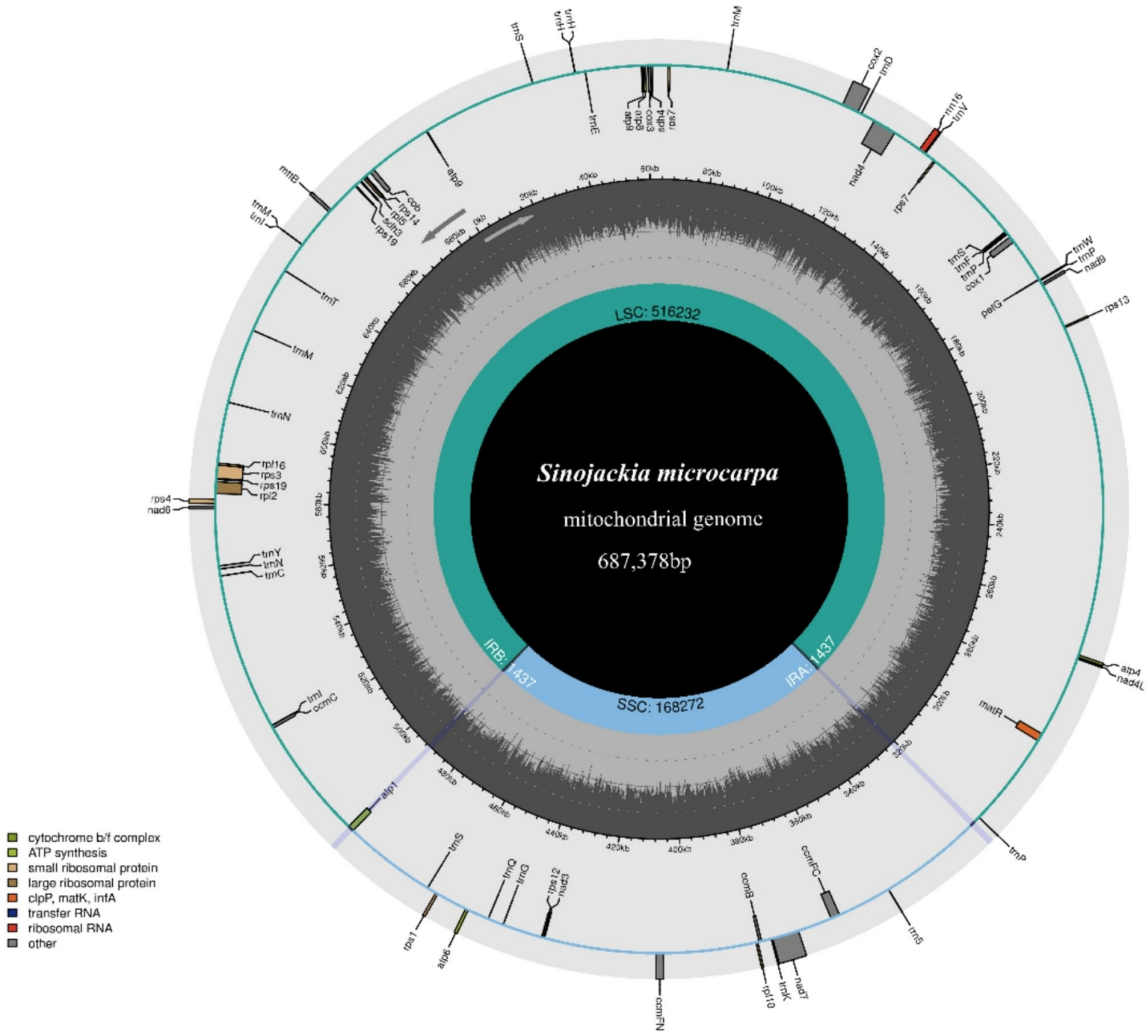
Circular mitochondrial genome of *S. microcarpa*. Genes belonging to different functional groups are color-coded. The GC content is represented on the inner circle by the dark gray plot

The *S. microcarpa* mitochondrial genome was assembled into a single, large circular molecule, with a total length of 687,378 bp and an overall GC content of 46.24% (GenBank accession: OL693656) (Fig. 1). A total of 59 genes were identified, comprising 37 protein-coding genes (PCGs), 2 ribosomal RNA (rRNA) genes, and 20 tRNA genes, all of which were predicted and annotated using BLAST and tRNAscan-SE tools. (Table 1 and Supplemental S1). The noncoding sequence is 654,741 bp in size and accounts for 95.25% of the whole-genome sequence, which is much greater than the average noncoding sequence content (89.46%) in other angiosperms [34]. Among the 37 PCGs that are conserved among all plant mitochondrial genomes, 15 genes encode electron transport proteins and ATP synthase, including six subunits of complex I (*nad3*,* 4*,* 4 l*,* 6*,* 7* and *9*), one subunit of complex III (*cob*), three subunits of complex IV (*cox1*, *cox2* and *cox3*), and five subunits of complex V (*atp1*, *4*,* 6*,* 8*, and 9) (Supplemental Table S1). Furthermore, there were four genes for large ribosomal proteins (*rpl2*,* 5*,* 11*, and *16*), eight genes for small ribosomal proteins (*rps1*,* 3*,* 4*,* 7*,* 12*,* 13*,* 16*, and *19*), four genes for cytochrome c biogenesis (*ccmB*, *ccmC*, *ccmFN*, and *ccmFC*) and two genes for the maturase and transporter (*matR* and *matB*).

**Table 1:** The list of genes in the mitochondrial genome of *S. microcarpa*

Gene group	Gene
Complex I	nad3 nad4 nad4L nad6 nad7 nad9^*^
Complex III	cob
Complex IV	cox1 cox2 cox3
Complex V	atp1 atp4 atp6 atp8 atp9
Succinate dehydrogenase	sdh3 sdh4
Ribosomal large subunit	rpl10 rpl16 rpl2 rpl5
Ribosomal small subunit	rps1 rps12 rps13 rps14 rps19 rps3 rps4 rps7^*^
Cytochrome C	cox1 cox2 cox3
others	matR mttB petG
Ribosomal RNAs	rrn16 rrn5
Transfer RNAs	trnE-TTC trnS-GCT trnF-GAA trnP-TGG^+^ trnG-GCC trnQ-TTG trnC-GCA trnN-GTT trnY-GTA trnM-CAT^+^ trnK-TTT trnW-CCA trnV-GAC trnD-GTC trnH-GTG trnS-TGA

*, the gene has two copies in the genome; +, the gene has 3 copies in the genome

The total length of the 37 protein-coding genes (PCGs), ranging from 225 bp (*atp9*) to 1,968 bp (*mat-R*), was 23,139 bp, representing 3.37% of the mitochondrial genome. Additionally, six intron-containing genes were identified, including *nad4*, *nad7*, *cox2*, *rpl12*, *ccmFC*, and *rps3*. Of the tRNA genes, 12 were of mitochondrial origin, while eight were derived from plastids. A total of 117 open reading frames (ORFs) larger than 50 amino acids were also detected using ORF-Finder. Most open reading frames (ORFs) contain a single gene copy ranging from 150 to 1,000 bp, with only two exceeding 1,000 bp. Despite the high number of ORFs identified, none could be assigned specific functions based on protein sequence similarity. Among the 37 core protein-coding genes, most were identical to those found in the mitochondrial genome of *Stewartia sinensis*, except for *nad1*, *nad2*, and *nad5*, which were absent in *S. microcarpa*.

### Repeat sequences in the S. microcarpa mitochondrial genome

Repetitive sequences are widespread in the mitochondrial genomes of terrestrial plants and present substantial polymorphism. These sequences frequently involve numerous genomic rearrangements, resulting in diverse stoichiometries [29, 35]. A total of 50 pairs of large repetitive sequences ranging from 78 to 4256 bp in size were identified in the *S. microcarpa* mitochondrial genome (Table 2). Most of these repeats were either forward or palindromic. Of the identified repeats, three (R1–R3) exceeded 1,000 bp in length, while 32 repeats (R4–R35) ranged between 100 and 999 bp. The remaining 16 repeats (R35–R50) measured between 70 and 99 bp. Each of these large repeat sequences was present in two copies. Additionally, 27 tandem repeats longer than 10 bp were detected in the *S. microcarpa* mitochondrial genome. (Supplemental Table S2). The length of the repeat units in these regions varied between 11 and 48 bp, with an identity percentage ranging from 80 to 100%. In total, these tandem and scattered repeat sequences accounted for 0.2% of the *S. microcarpa* mitochondrial genome.

**Table 2:** Large repeats in the *S. microcarpa* mitochondrial genome

		*Copy 1*		*Copy 2*			
**No.**	**Size (bp)**	**Start**	**End**	**Start**	**End**	**Repeat distance**	**Type**
R1	4256	632,200	636,455	682,131	686,386	0	F
R2	2948	0	2947	56,030	58,977	0	F
R3	1437	320,261	321,697	489,970	491,406	0	P
R4	999	55,031	56,029	686,379	687,377	0	F
R5	768	6777	7544	344,722	345,489	-1	P
R6	762	6783	7544	344,722	345,483	0	P
R7	331	585,861	586,191	667,771	668,101	-2	F
R8	317	585,875	586,191	667,785	668,101	-1	F
R9	226	393,479	393,704	518,976	519,201	0	P
R10	212	375,914	376,125	566,015	566,226	0	P
R11	221	6725	6945	109,191	109,411	-3	P
R12	207	585,985	586,191	667,895	668,101	0	F
R13	208	6738	6945	109,191	109,398	-2	P
R14	201	6745	6945	109,191	109,391	-1	P
R15	197	110,270	110,466	188,079	188,275	-2	P
R16	169	109,191	109,359	345,321	345,489	-2	F
R17	162	596,042	596,203	667,549	667,710	-1	F
R18	152	109,208	109,359	345,338	345,489	-1	F
R19	136	96,867	97,002	483,837	483,972	0	P
R20	138	322,666	322,803	580,839	580,976	-3	F
R21	132	322,672	322,803	580,845	580,976	-2	F
R22	122	346,333	346,454	578,651	578,772	-1	F
R23	115	398,653	398,767	511,178	511,292	0	P
R24	113	185,579	185,691	593,837	593,949	0	F
R25	111	29,415	29,525	349,850	349,960	0	F
R26	111	44,913	45,023	100,400	100,510	0	P
R27	113	33,916	34,028	347,704	347,816	-1	P
R28	108	608,749	608,856	632,137	632,244	0	P
R29	103	38,077	38,179	436,252	436,354	0	P
R30	113	453,305	453,417	517,754	517,866	-3	P
R31	108	346,665	346,772	582,202	582,309	-3	P
R32	104	170,315	170,418	567,427	567,530	-2	P
R33	107	58,999	59,105	456,524	456,630	-3	P
R34	104	346,674	346,777	582,197	582,300	-3	P
R35	104	453,320	453,423	517,748	517,851	-3	P
R36	92	469,277	469,368	663,829	663,920	0	P
R37	94	453,330	453,423	517,748	517,841	-2	P
R38	86	597,102	597,187	670,114	670,199	0	F
R39	85	322,719	322,803	580,892	580,976	0	F
R40	84	205,498	205,581	413,026	413,109	0	P
R41	86	453,338	453,423	517,748	517,833	-1	P
R42	88	346,450	346,537	579,179	579,266	-2	F
R43	79	140,091	140,169	398,781	398,859	0	F
R44	78	218,236	218,313	573,526	573,603	0	P
R45	77	161,760	161,836	346,432	346,508	0	P
R46	77	244,144	244,220	306,854	306,930	0	F
R47	83	381,556	381,638	575,680	575,762	-2	F
R48	82	29,428	29,509	189,335	189,416	-2	F
R49	82	189,335	189,416	349,863	349,944	-2	F
R50	78	434,571	434,648	667,678	667,755	-1	F

Note. F, forward repeats; P, palindromic repeats

### Plastid-like sequences identified in the S. microcarpa mitochondrial genome

Sequencing analyses of the nuclear, mitochondrial, and chloroplast genomes have shown that genes can move between different genomes inside a cell [36]. There has been an upsurge in the publication and analysis of data relevant to organelle genomes due to the introduction of novel methods [23, 37]. It is believed that long-term plant evolution includes gene transfer between chloroplasts and mitochondria [38, 39]. In this study, we employed BLASTn to detect sequences within the *S. microcarpa* mitochondrial genome that are homologous to plastid DNA. We successfully identified 16 sequence fragments that demonstrated greater than 90% nucleotide sequence identity to the corresponding plastid sequences of *S. microcarpa*. The identified plastid-like sequences varied in size from 28 to 468 base pairs and collectively amounted to a total length of 1785 base pairs, representing 0.26% of the entire mitochondrial genome (Table 3). Within this array, nine genes related to tRNA and six partial protein-coding genes (PCGs) were associated primarily with photosynthesis, transcription, and translation processes. Additionally, an intact plastid-derived gene, rps7, was identified in the mitochondrial genome of *S. microcarpa*. Collectively, these findings substantiate the prevalence of intracellular DNA transfer from plastids to mitochondria in *S. microcarpa*.

**Table 3:** Distribution of plastid-derived sequences in the *S. microcarpa* mitochondrial genome

Sequence	Position	gene
cp1	42,119–42,193	trnH-cp
cp2	114,660–114,733	trnD-cp
cp3	133,231–133,302	trnV-cp
cp4	135,158–135,380	partial rps12
cp5	136,064–136,472	rps7
cp6	174,499–175,571	trnW-cp
cp7	175,266–175,379	partial petG
cp8	175,731–175,804	trnP-cp
cp9	380,549–380,576	partial atpB
cp10	522,820–522,895	trnI-cp
cp11	522,820–522,895	trnI-cp
cp12	554,148–554,281	partial rbcL
cp13	605,135–605,182	trnN-cp
cp14	613,308–613,338	partial ndhJ
cp15	623,869–623,940	trnM-cp
cp16	626,934–627,080	partial psbC
cp14	613,308–613,338	partial ndhJ
cp15	623,869–623,940	trnM-cp
cp16	626,934–627,080	partial psbC

While the intercompartmental transfer of DNA sequences from plastids to mitochondria is a widespread phenomenon across terrestrial plant species, there is considerable variation in the extent and magnitude of these transferred sequences among different taxa. In *S. microcarpa*, a mere 0.26% of the mitochondrial genome comprises sequences of plastid origin, a proportion markedly less than the 3–6% plastid DNA typically found within the mitochondrial genomes of other plant species [23]. The reason for this might be the relatively larger size of the *S. microcarpa* mitochondrial genome. Moreover, in the plastid-derived sequences, most were partial sequences that showed no functionality except for some tRNA genes, which is consistent with other land plants [40]. In *S. microcarpa*, an intact plastid-derived rps7 gene was identified in the mitochondrial genome, which is also frequently found in most angiosperms [23]. By studying the genomes of plant chloroplasts and mitochondria, additional insights into the evolution of phylogenetic analyses and molecular markers of these specially arranged genomes can be obtained.

### Relative synonymous codon usage of the S. microcarpa mitochondrial genome

**Fig. 2:**
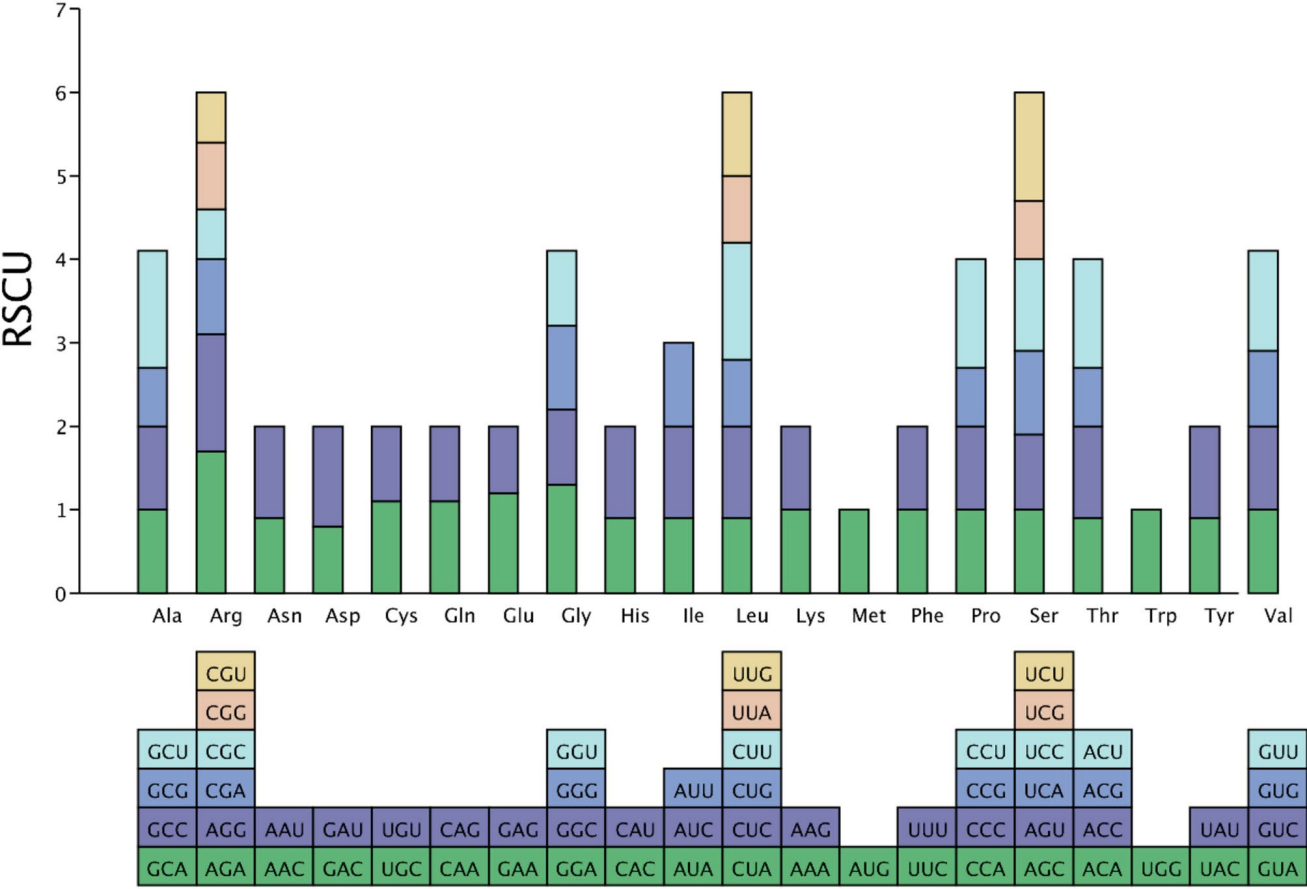
Relative synonymous codon usage. Different proportions correspond to different RSCUs

Codon usage bias is thought to result from a relative balance within the cell over a long period of evolutionary selection [41–43]. An RSCU value greater than 1 was considered to indicate the beneficial effect of amino acids. There were 29 codons for which the RSCU > 1 (Fig. 2). The RSCU values of the start codons AUG phenylalanine (UUC) (UUU) and tryptophan (UGG) were one. The remaining mitochondrial protein-coding genes exhibited general codon usage (Fig. 2). The stop codon showed no significant bias, with all three termination codons being approximately 1. Arginine (Arg) had the highest preference for AGA codons, with an RSCU value of 1.68 (Fig. 2). Notably, cysteine (Cys) and phenylalanine (Phe) did not have strong codon usage, with maximum RSCU values less than 1.1 (Fig. 2). The results revealed that A or T nucleotides were used more frequently at the third codon position than were C or G nucleotides. This result supports the widespread phenomenon that A/T nucleotides are used more frequently at the third codon position than C/G nucleotides. The underlying reasons for this are closely related to natural selection and mutational pressure [44–46].

### The Non-synonymous (Ka)/synonymous (Ks) in the *S. microcarpa* mitochondrial genome

**Fig. 3:**
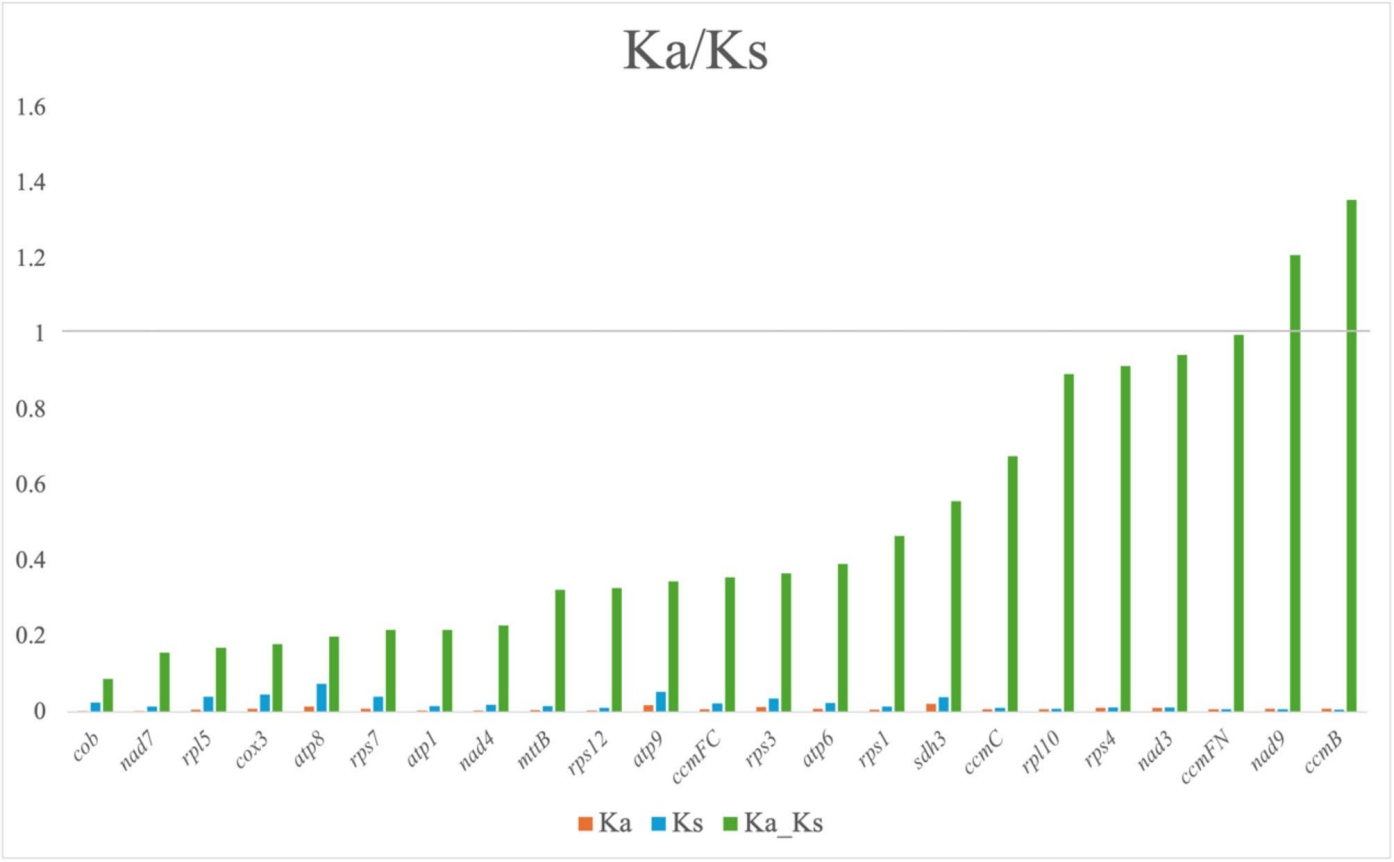
The Non-synonymous (Ka)/synonymous (Ks) ratio values for 23 mitochondrial protein-coding genes of *Sinojackia microcarpa* compared with the mitochondrial genome of *Stewartia sinensis* Ka represents the non-synonymous substitution rate, Ks represents the synonymous substitution rate, and Ka/Ks indicates the selective pressure acting on each gene

The Ka/Ks ratio, which compares the rate of non-synonymous substitutions (Ka) to synonymous substitutions (Ks), serves as an indicator of selective pressures acting on protein-coding genes [47]. Based on our comparative analysis, we calculated the Ka/Ks ratios for 23 mitochondrial protein-coding genes with *Stewartia sinensis* to evaluate the selective pressures acting on these genes (Fig. 3, Table S3). Specifically, the majority of the analyzed genes (e.g., *cob*, *nad7*, *cox3*, *atp8*, *rps7*, and *nad4*) showed Ka/Ks ratios significantly lower than 1, indicating strong evolutionary constraints and high functional conservation. This aligns with the expectation that mitochondrial genes are typically conserved to maintain essential physiological functions. Notably, two genes—*nad9* (Ka/Ks = 1.21) and *ccmB* (Ka/Ks = 1.36)—exhibited Ka/Ks ratios greater than 1, suggesting that these genes may be undergoing positive selection, possibly related to environmental adaptation or functional divergence. These unusual evolutionary patterns merit further investigation through functional experiments and ecological analyses, to clarify the adaptive roles of *nad9* and *ccmB* genes in future studies.

In conclusion, the Ka/Ks analysis reinforces the evolutionary conservation of mitochondrial functions while identifying specific genes that might be undergoing adaptive evolution. Further research could focus on understanding the biological significance of these genes and their potential role in the plant’s evolutionary adaptability.

### Conserved gene clusters in the *S. microcarpa* mitochondrial genome

**Fig. 4:**
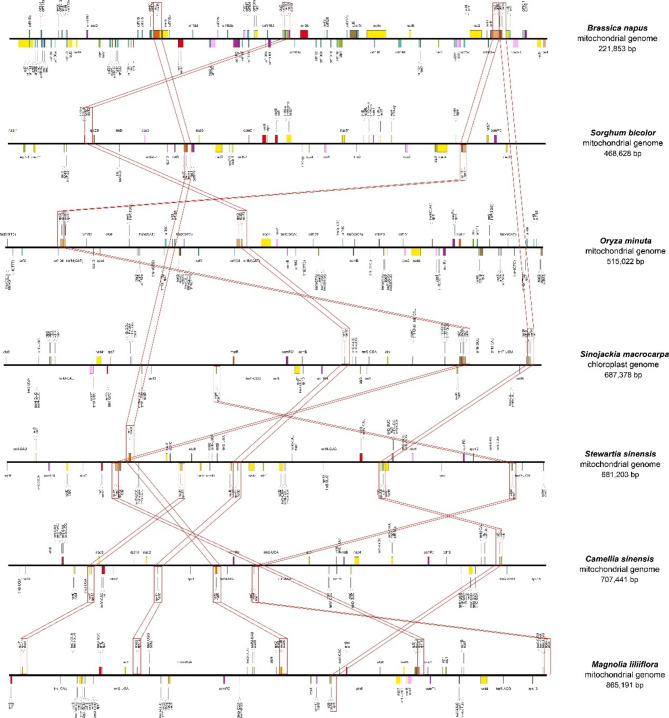
Analysis of conservative gene clusters between the *S. microcarpa* mt genome and other plant mt genomes

Plant mitochondrial genomes exhibit high variability in gene organization due to homologous recombination, sequence duplications, genome expansion and contraction, and foreign DNA incorporation despite a low point mutation rate [48, 49]. Recombination can disrupt gene clusters and, through multiple events, generate similar gene clusters, causing significant differences in gene order [38, 39]. In this study, we compared gene order across the mitochondrial genomes of seven angiosperm species, including four monocots and three dicots, and quantified the number of syntenic gene clusters, defined as genes maintaining their relative order. The results revealed that the rps12-nad3 gene cluster was conserved across all seven mitochondrial genomes (Fig. 4; Supplemental Table S4), indicating its high level of conservation in plant mitochondrial genomes. Additionally, the *rps3-rpl16* cluster (absent in *Camellia sinensis*), *nad1-matR* (absent in *S. microcarpa* and *Oryza minuta*), and *rpl5-rps14* (absent in *Sorghum bicolor* and *Oryza minuta*) were retained in most species. The *nad1-rpl13* cluster (absent in *S. microcarpa*) and *atp4-nad4L* were predominantly found in Gramineae (monocots) but were either absent or lost in the three dicot species.

The variability in gene order across the species, particularly the disruption of gene clusters due to homologous recombination, supports the hypothesis of a highly dynamic mitochondrial genome [48, 49]. The presence of different gene clusters in specific lineages, such as *S. microcarpa*, suggests that recombination events have shaped its mitochondrial genome, leading to the diversification of these gene arrangements. These differences reflect evolutionary pressures that vary across species and might be influenced by factors like genome size, environmental adaptation, and evolutionary history [19, 50]. The rps12-nad3 cluster’s conservation across all species studied indicates its fundamental role in mitochondrial function, likely related to the electron transport chain and energy metabolism. Other conserved clusters, such as rps3-rpl16 and nad1-matR, are essential for protein synthesis and regulation, which are critical functions in maintaining mitochondrial integrity and overall cellular function. In our study, the gene clusters in the *S. microcarpa* mitochondrial genome appear to be mostly consistent with those of dicot, indicating the conservation of the gene orders during the evolution of dicot species.

### Distribution of S. microcarpa tRNAs compared with those of other species

**Fig. 5:**
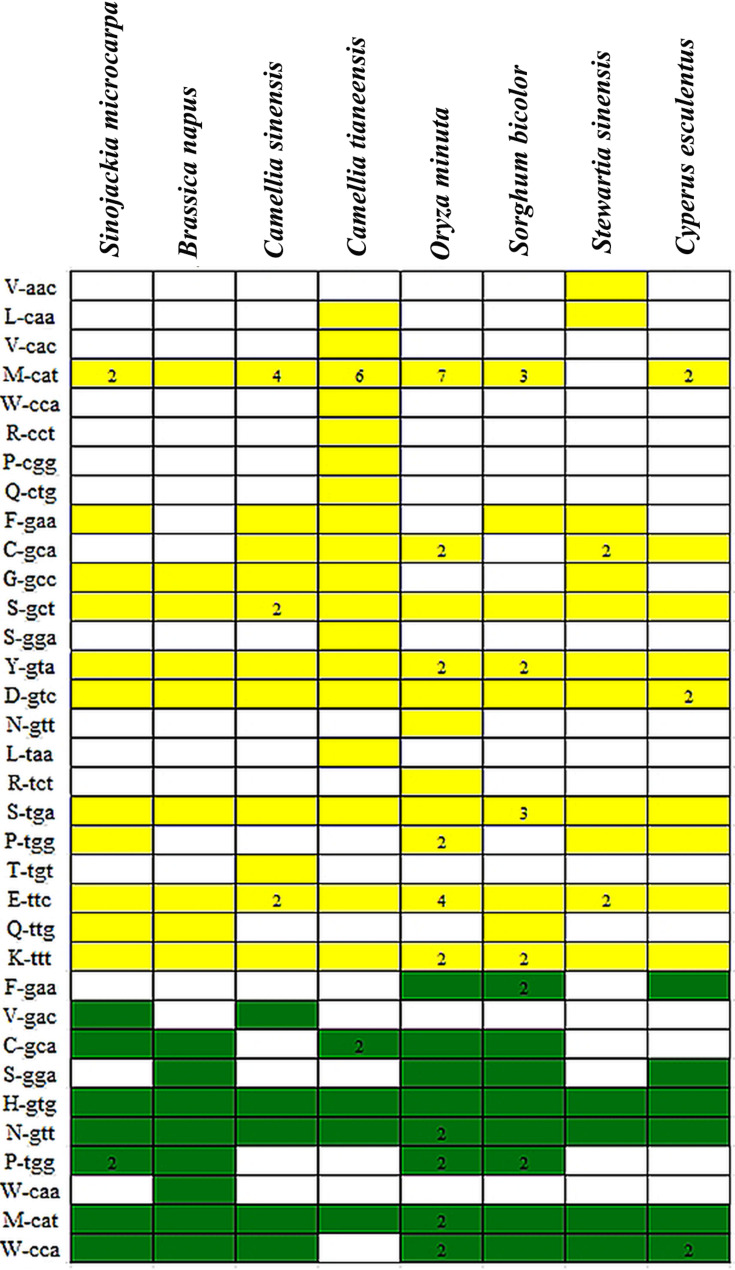
Distribution of transfer RNA (tRNA) genes in plant mitochondrial genomes. The white boxes indicate that the gene is absent or lost in the mitochondrial genome. Yellow and green boxes indicate mitochondrial tRNA genes and plastid-derived tRNA genes, respectively, with one copy existing in each mitochondrial genome. The numbers represent the copy numbers in the mitochondrial genome

A complete set of tRNAs is essential for protein translation within the plant mitochondrial genome. However, during the evolutionary process in higher plants, many tRNA genes may be lost, relocated, or inactivated [48, 49, 51]. To examine the origin and distribution of these tRNA genes, tRNAscan-SE 2.0 was employed to predict the tRNAs in the *S. microcarpa* mitochondrial genome. Out of the 20 tRNA genes identified, 12 were of mitochondrial origin, while eight were plastid-derived (Fig. 5) [52]. A comparative analysis involving seven other monocot and dicot species indicated that certain mitochondrial tRNAs, including *trnS*, *trnE*, *trnY*, *trnD*, and *trnK*, were consistently present in all species, suggesting their evolutionary conservation in plant mitochondrial genomes. Conversely, other mitochondrial tRNA genes exhibited more variable distributions. Notably, *trnL* was absent in all monocots and dicots except *Camellia tianeensis*. Among the plastid-originated tRNAs, *trnM*, *trnH*, and *trnN* were conserved across all examined mitochondrial genomes, highlighting their phylogenetic significance [53–55]. TrnL was absent in all monocotyledonary and dicotyledonary species, except for *Camellia tianeensis*. Among the plastid-like tRNAs, three tRNAs (trnM, trnH, and trnN) were conserved in all the analyzed mitochondrial genomes.

**Fig. 6:**
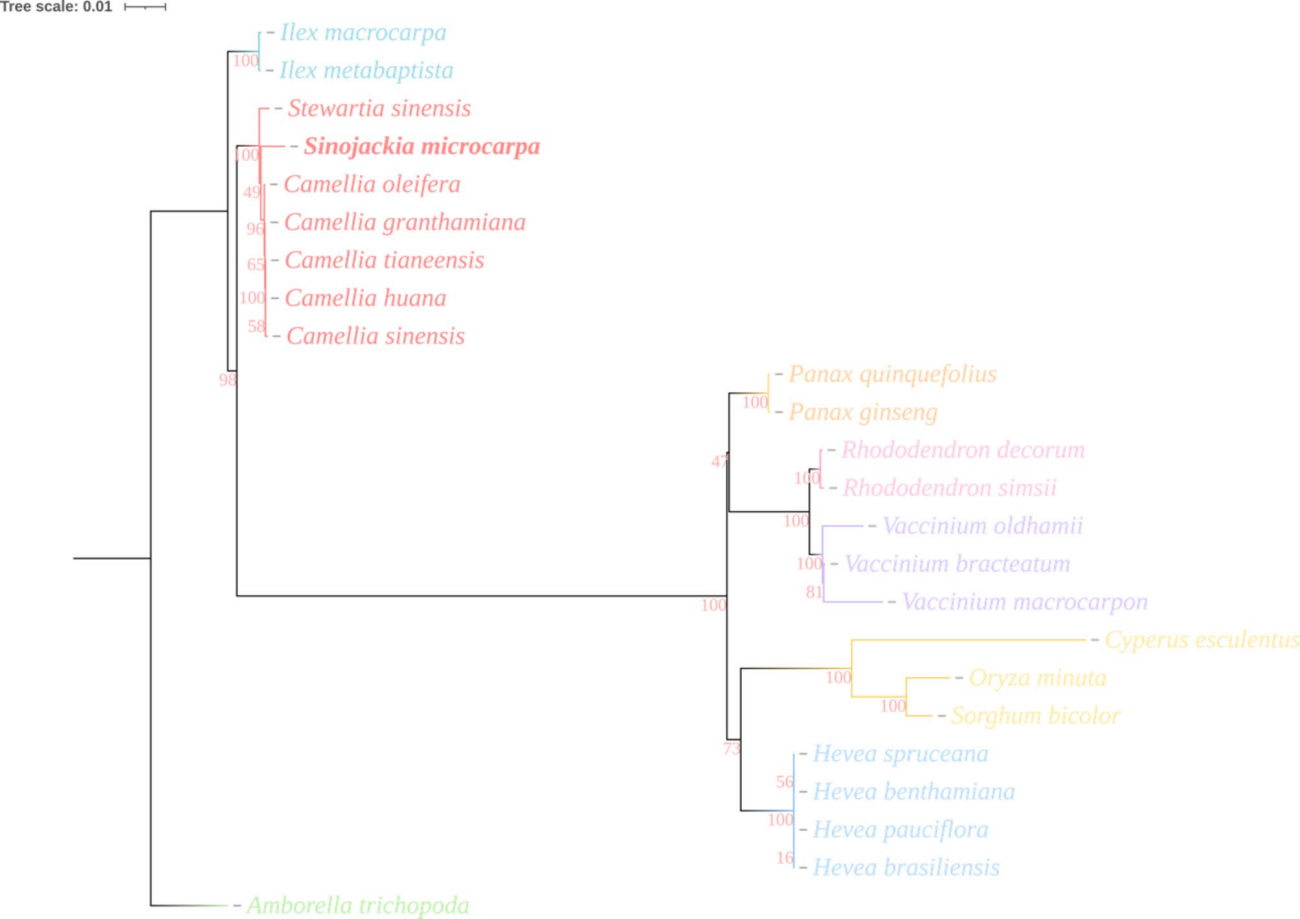
Phylogenetic tree based on 29 homologous protein-coding genes in the mitochondrial genomes of 24 plants via maximum likelihood (ML) analysis. The numbers below the nodes are support values with ML bootstrap values. *Amborella trichopoda* was designated as the outgroup. Scale bar represents nucleotide substitutions per site

### Phylogenetic analysis of S. microcarpa

The maximum likelihood phylogenetic tree, constructed using twenty-nine homologous mitochondrial protein-coding genes from twenty-four plant species, provides a framework for understanding the evolutionary relationships among these taxa. At the root of the tree, basal angiosperms such as *Amborella trichopoda* occupy the earliest diverging branch, establishing a foundational point that reflects the ancestral state of flowering plants.

*S. microcarpa*’s positioning in the tree places it among dicots, specifically within a cluster that includes *Stewartia* and *Camellia*, suggesting that *S. microcarpa* and these species share certain evolutionary traits, potentially related to mitochondrial genome structure or other genomic features that evolved together. The high bootstrap support values for these nodes indicate robust confidence in this grouping, implying that these species share a relatively recent common ancestor. Other genera, such as *Hevea* (rubber tree) and *Vaccinium* (blueberry), are placed further apart in the tree, indicating greater evolutionary divergence (Fig. 6).

## Discussion

The assembled mitochondrial genome of *S. microcarpa* is an essential resource that enhances our understanding of plant mitochondrial diversity and evolutionary dynamics [49]. This genome assembly helps fill a critical gap in the phylogenetic data available for the Styracaceae family, offering new insights into how mitochondrial genomes have evolved in terms of size, gene content, and structure [32].

One of the findings from this study is the significant presence of plastid-derived sequences within the mitochondrial genome [38–40]. The identification of sixteen plastid-like sequences, including an intact rps7 gene, highlights the dynamic nature of gene transfer between organelles. This intercompartmental gene transfer is not only pivotal in understanding organelle evolution but also raises questions about the role these genes may play in the adaptability and function of the mitochondria in *S. microcarpa*. Meanwhile, given the endangered status of *S. microcarpa*, the genetic information derived from its mitochondrial genome is vital for conservation efforts [20]. Understanding the genomic structure can help in developing strategies to monitor genetic variation and adaptability under environmental stressors, aiding in the species’ preservation and management [56]. Further studies should focus on comparative genomic analyses between *S. microcarpa* and other members of the Styracaceae family to better understand the evolutionary pressures that shaped their genomes [24]. Additionally, exploring the functional significance of the plastid-derived genes found in the mitochondrial genome could provide deeper insights into their roles in cellular metabolism and stress responses. Overall, the mitochondrial genome of *S. microcarpa* contributes to our understanding of mitochondrial genome evolution in plants and sets a foundation for future genetic studies.

Although we successfully assembled the mitochondrial genome of *S. microcarpa* using high-throughput sequencing technologies, we acknowledge certain limitations in the current assembly. Despite using multiple sequencing techniques, including Illumina and PacBio, the assembly is still constrained by sequencing depth and coverage. Specifically, low coverage in certain regions may result in incomplete assembly of genome fragments, potentially affecting the integrity and accuracy of the genome. The mitochondrial genome typically contains highly repetitive sequences and gene rearrangements, which present challenges for assembly. Although we made efforts to assemble the genome, the presence of repetitive sequences may have led to incomplete or inaccurate processing of these regions. As a result, some genes or regulatory regions might be missing or incomplete. This mitochondrial assembly is primarily based on the available sequencing data, and while comparisons with genomes of other species have enhanced the reliability of the assembly, some limitations of the assembly process remain. Therefore, the current assembly may not fully represent the entire complexity of the *S. microcarpa* mitochondrial genome. Future research could improve these limitations by increasing sequencing depth or employing more advanced assembly methods, as well as further validating gene functions.

## Materials and methods

### DNA sequencing and genome assembly

*Sinojackia microcarpa* Tao Chen & G. Y. Li was obtained from the Campus of Zhejiang A&F University Hangzhou, Zhejiang Province (30°15′52″N, 119°43′33″E), China [57]. The voucher specimens are available at the College of Forestry and Biotechnology, Zhejiang A&F University (Accession No. BR01). Total genomic DNA was extracted via a modified cetyltrimethylammonium bromide (CTAB) method and applied to 500-bp paired-end library construction via the NEBNext Ultra DNA Library Prep Kit for Illumina sequencing [58]. Sequencing was carried out on the Illumina NovaSeq 6000 platform (BIOZERON Co., Ltd., Shanghai, China). Approximately 3.66 Gb of raw data from *S. microcarpa* were generated with 150 bp paired-end read lengths. For the construction and sequencing of the PacBio library, more than 5 µg of sheared and concentrated DNA was subjected to size selection by Blue Pippin (Sage Science, Beverly MA, USA). Approximately 20 kb SMRTbell libraries were prepared according to the manufacturer’s instructions (PacBio, Menlo Park, CA, USA). A total of 102.6 million reads were sequenced on the PacBio Sequel platform.

De novo assembly via NOVOPlasty referencing the mt genomes of closely related species (GenBank Acc. No. NC_016004.1, NC_016005.1 and NC_016006.1)) produced 165 scaffolds in the mt genome. BLAST searches against the mitochondrial genomes of related species, such as Cucumis sativus and NOVOPlasty, revealed that a small number of possible mitochondrial reads were isolated from the pool of Illumina data. The SPAdes-3.13.0 package was used to acquire the mitochondrial Illumina reads for the purpose of performing mt genome de novo assembly [59]. Clean PacBio long reads were aligned against the NOVOPlasty- and SPAdes-assembled scaffolds via the BWA men program [60]. The Canu v2.1.1 package was used to perform self-correction and mt genome de novo assembly on all the aligned PacBio reads. Racon v1.4.3 and Pilon v1.21 were subsequently used to rectify errors [61]. The overlap and connectivity between the sequences that PacBio had assembled were then examined.

### Annotation and comparison of the mitochondrial DNA sequences

The mitochondrial genes were annotated via the online GeSeq tool, with default parameters used to predict protein-coding genes and ribosome RNA (rRNA) genes [62]. Transfer RNA (tRNA) genes were identified via tRNAScan-SE 2.0 software (http://lowelab.ucsc.edu/tRNAscan-SE/) [52]. MEGA 11 was used to analyze basic sequence information such as base composition, nucleotide sequence information sites, start codons, and stop codons and to calculate relative synonymous codon usage (RSCU). The results were visualized via the R package ggplot2 [63].

Open reading frames (ORFs) that contained > 50 amino acid residues starting with methionine were predicted and annotated via ORF-Finder (http://www.ncbi.nlm.nih.gov/gorf/gorf.html) [64]. The repeat sequences were analyzed via REPuter software (http://bibiserv.techfak.unibielefeld.de/reputer) with the following parameters: the repeat sequence was at least 50 bp in length, and the repeat identity was > 90% [65]. The circular map and syntenic gene cluster maps of the plant mitochondrial genomes were created via Chloroplot (https://irscope.shinyapps.io/chloroplot/) [66].

### Phylogenetic analysis

To compare the *S. microcarpa* mitochondrial genome with those of other plant mitochondrial genomes, twenty-three plant species, including *Camellia sinensis* (NC_043914.1), *Cyperus esculentus* (NC_058697.1), *Panax ginseng* (MZ389476.1), *Panax quinquefolius* (NC_067574.1), *Hevea spruceana* (NC_084328.1), *Hevea brasiliensis* (AP014526.1), *Hevea benthamiana* (OR663908.1), *Hevea pauciflora* (NC_080334.1), *Vaccinium oldhamii* (PP812361.1), *Vaccinium macrocarpon* (NC_023338.1), *Vaccinium bracteatum* (PQ283854.1), *Ilex macrocarpa* (NC_082235.1), *Ilex metabaptista* (NC_081509.1), *Camellia granthamiana* (NC_086761.1), *Camellia oleifera* (PQ557234.1), *Camellia huana* (PP975887.1), *Camellia tianeensis* (PP727208.1), *Rhododendron decorum* (NC_073150.1), *Rhododendron simsii* (NC_053763.1), *Oryza minuta* (NC_029816.1), *Sorghum bicolor* (NC_008360.1), *Amborella trichopoda* (KF754799.1 ~ KF754803.1) and *Stewartia sinensis* (NC_081928.1). Twenty-four protein-coding genes (PCGs) (*atp1*,*atp4*,*atp6*,*atp8*,*atp9*,*ccmB*,* ccmC*,* ccmFc*,* ccmFn*,* cob*,* cox3*,*mttB*,* matR*,* nad1*,*nad3*,*nad4*,*nad4*,*nad6*,*nad7*,*nad9*,*rpl10*,*rpl16*,*rpl5*,*rps1*,*rps3*,*rps4*,*rps7*,*rps12*,*rps19)* from each mitochondrial genome were aligned via ClustalW and manually edited to remove gaps and missing data [67]. Phylogenetic analysis was performed via MEGA 11 software with 1,000 bootstrap replicates via the best-fit model [68]. The maximum likelihood method was used to construct the original phylogenetic trees.

## Conclusions

In this study, we sequenced and annotated the complete mitochondrial genome of *S. microcarpa* for the first time. The genome spans 687,378 bp with a glycine–cysteine (GC) content of 46.24%. A total of 59 genes were predicted, including 37 protein-coding genes (PCGs), 2 ribosomal RNA (rRNA) genes, and 20 transfer RNA (tRNA) genes. Remarkably, the noncoding regions constituted 95.25% of the genome, which is significantly higher than in other angiosperms. Additionally, we identified 16 sequences resembling those from plastids, including a fully intact plastid-related gene, *rps7*. Codon usage analysis demonstrated a preference for A or T nucleotides at the third codon position over C or G nucleotides. The phylogenetic analysis of 29 protein-coding genes across 24 plant species provides valuable insights into the evolutionary relationships within the plant kingdom, particularly concerning *S. microcarpa*. The placement of *S. microcarpa* close to species from the *Camellia* genus suggests a relatively recent common ancestor.

## Electronic supplementary material

Below is the link to the electronic supplementary material.

**Supplementary Material 1**: **Additional files**: **Table S1.** Summary of *Sinojackia microcarpa* mitochondrial genome features. **Table S2.** Distribution of tandem repeats in the *Sinojackia microcarpa* mitochondrial genome. **Table S3.** Pairwise nonsynonymous (dN)/synonymous (dS) substitution rates among mitochondrial genes of *Sinojackia microcarpa* and *Stewartia sinensis*. **Table S4.** Distribution of gene clusters in the mitochondrial genomes of land plants.

## Data Availability

The data that support the findings of this study have been deposited in the NCBI database [GenBank accession: OL 693656] (http://www.ncbi.nlm.nih.gov/).

## Abbreviations

rRNA: Ribosomal RNA
tRNA: Transfer RNA
PCGs: Protein-coding genes
ORF: Open reading frame
CTAB: Cetyltrimethylammonium bromide
RSCU: Relative synonymous codon usage ratios
ML: Maximum Likelihood
