# Supplementary material for: The complete mitochondrial genome of Sinojackia microcarpa: evolutionary insights and gene transfer
Source: BMC Genomics. 2025 May 6;26:446. doi: 10.1186/s12864-025-11633-7 (PMC12054226; doi:10.1186/s12864-025-11633-7)
Supplement: Supplementary file 1 — Supplementary Material 1: Additional files: Table S1. Summary of Sinojackia microcarpa mitochondrial genome features. Table S2. Distribution of tandem repeats in the Sinojackia microcarpa mitochondrial genome. Table S3. Pairwise nonsynonymous (dN)/synonymous (dS) substitution rates among mitochondrial genes of Sinojackia microcarpa and Stewartia sinensis. Table S4. Distribution of gene clusters in the mitochondrial genomes of land plants. [file 12864_2025_11633_MOESM1_ESM.docx]

**Table S1** Summary of *Sinojackia* *microcarpa* mitochondrial genome features

| **Category** | **Feature** | **Number** | **Size（bp,%）** |
| --- | --- | --- | --- |
| **Genome** | G+C | - | 317814(46.24) |
| **Genes (total)** | Protein coding | 37 | 29,102(4.23） |
|  | rRNA | 2 | 1609(0.23) |
|  | tRNA | 20 | 1926(0.28) |
|  | Open reading frame | 117 | 32961(4.80) |
| **Repeats** | Large repeats (>1,000) | 3 | 8641(1.26) |
|  | Medium repeats (>100, <999) | 32 | 7074(1.03) |
|  | Short repeats (>70, <99) | 15 | 1251(0.18) |

**Table S2.** Distribution of tandem repeats in the *Sinojackia microcarpa* mitochondrial genome

| **Indices** | **Period Size** | **Copy Number** | **Consensus Size** | **Percent Matches** |
| --- | --- | --- | --- | --- |
| 51731--51780 | 25 | 2 | 25 | 92 |
| 81607--81649 | 18 | 2.4 | 18 | 80 |
| 98140--98169 | 15 | 2 | 15 | 100 |
| 107787--107824 | 17 | 2.2 | 17 | 90 |
| 134187--134224 | 18 | 2.1 | 18 | 90 |
| 134712--134761 | 21 | 2.3 | 22 | 83 |
| 134737--134767 | 15 | 2.1 | 15 | 93 |
| 136938--136971 | 17 | 2 | 17 | 100 |
| 207843--207885 | 17 | 2.4 | 18 | 80 |
| 278994--279031 | 16 | 2.4 | 16 | 91 |
| 281463--281534 | 33 | 2.2 | 32 | 85 |
| 283025--283064 | 21 | 1.9 | 21 | 89 |
| 317807--317837 | 16 | 1.9 | 16 | 93 |
| 346027--346083 | 18 | 3.2 | 18 | 97 |
| 391451--391484 | 16 | 2.1 | 17 | 94 |
| 401573--401667 | 48 | 2 | 48 | 97 |
| 421880--421908 | 14 | 2.1 | 14 | 100 |
| 434223--434252 | 15 | 2 | 15 | 100 |
| 434708--434871 | 45 | 3.6 | 45 | 87 |
| 466110--466139 | 11 | 2.6 | 11 | 94 |
| 480541--480575 | 16 | 2.2 | 16 | 89 |
| 512373--512422 | 21 | 2.4 | 21 | 82 |
| 536007--536114 | 36 | 3 | 36 | 100 |
| 601749--601807 | 31 | 1.9 | 30 | 82 |
| 602392--602422 | 14 | 2.2 | 14 | 94 |
| 627877--627955 | 39 | 2 | 39 | 97 |
| 642667--642742 | 38 | 2 | 38 | 89 |

**Table S3.** Pairwise nonsynonymous (Ka)/synonymous(Ks)substitution rates among mitochondrial genes of *Sinojackia microcarpa* and *Stewartia sinensis*

| Seq_1 | Seq_2 | Ka | Ks | Ka/Ks |
| --- | --- | --- | --- | --- |
| *sm.atp9* | *ss.atp9* | 0.018519459 | 0.053594223 | 0.34554955 |
| *sm.atp8* | *ss.atp8* | 0.014949532 | 0.074750614 | 0.1999921 |
| *sm.cox3* | *ss.cox3* | 0.008422323 | 0.046890268 | 0.17961772 |
| *sm.rps7* | *ss.rps7* | 0.008810674 | 0.040482001 | 0.21764423 |
| *sm.nad4* | *ss.nad4* | 0.004470286 | 0.019526905 | 0.22892957 |
| *sm.nad9* | *ss.nad9* | 0.009193756 | 0.007599812 | 1.20973473 |
| *sm.ccmFC* | *ss.ccmFC* | 0.008050392 | 0.022564151 | 0.35677795 |
| *sm.nad7* | *ss.nad7* | 0.002232145 | 0.01418482 | 0.1573615 |
| *sm.ccmB* | *ss.ccmB* | 0.008713113 | 0.006427463 | 1.35560688 |
| *sm.rpl10* | *ss.rpl10* | 0.008057374 | 0.009009117 | 0.89435774 |
| *sm.ccmFN* | *ss.ccmFN* | 0.006961953 | 0.006972741 | 0.99845289 |
| *sm.nad3* | *ss.nad3* | 0.011229155 | 0.011881437 | 0.94510082 |
| *sm.rps12* | *ss.rps12* | 0.003554509 | 0.010830513 | 0.32819396 |
| *sm.atp6* | *ss.atp6* | 0.009196927 | 0.023485285 | 0.39160381 |
| *sm.rps1* | *ss.rps1* | 0.006557419 | 0.014068408 | 0.46610952 |
| *sm.atp1* | *ss.atp1* | 0.003491677 | 0.016011282 | 0.21807606 |
| *sm.ccmC* | *ss.ccmC* | 0.007178162 | 0.010596203 | 0.67742773 |
| *sm.rps4* | *ss.rps4* | 0.011209342 | 0.012241006 | 0.91572064 |
| *sm.rps3* | *ss.rps3* | 0.013272955 | 0.036136021 | 0.36730539 |
| *sm.mttB* | *ss.mttB* | 0.00507831 | 0.015686846 | 0.32373044 |
| *sm.sdh3* | *ss.sdh3* | 0.021979595 | 0.039396365 | 0.55790922 |
| *sm.rpl5* | *ss.rpl5* | 0.006927123 | 0.040798637 | 0.16978809 |
| *sm.cob* | *ss.cob* | 0.002229242 | 0.025380065 | 0.08783436 |

sm: *Sinojackia microcarpa*; ss: *Stewartia sinensis.*

**Table S4.** Distribution of gene clusters in the mitochondrial genomes of land plants

| Species | *nad1-matR* | *rps12-nad3* | *rps3-rpl16* | *atp4-nad4L* | *nad1-rpl13* | *rpl5-rps14* |
| --- | --- | --- | --- | --- | --- | --- |
| *S. microcarpa* | - | + | + | + | - | + |
| *C. sinensis* | - | + | **/** | + | + | + |
| *S. sinensis* | + | + | + | + | + | + |
| *M. liliiflora* | + | + | + | + | + | + |
| *S. bicolor* | + | + | + | - | - | / |
| *O. minuta* | + | + | + | - | - | / |
| *B. napus* | + | + | + | - | - | + |

**+** indicates that the gene cluster exists in the plant mitochondrial genome; **-** indicates that the gene cluster is absent in the plant mitochondrial genome; **/** indicates that the gene cluster was lost.
